# Magnon spectrum of the helimagnetic insulator Cu_2_OSeO_3_

**DOI:** 10.1038/ncomms10725

**Published:** 2016-02-25

**Authors:** P. Y. Portnichenko, J. Romhányi, Y. A. Onykiienko, A. Henschel, M. Schmidt, A. S. Cameron, M. A. Surmach, J. A. Lim, J. T. Park, A. Schneidewind, D. L. Abernathy, H. Rosner, Jeroen van den Brink, D. S. Inosov

**Affiliations:** 1 Institut für Festkörperphysik, TU Dresden, Helmholtzstraße 10, D-01069 Dresden, Germany; 2 Max Planck Institute for Solid State Research, Heisenbergstraße 1, D-70569 Stuttgart, Germany; 3 Max Planck Institute for Chemical Physics of Solids, Nöthnitzer Straße 40, D-01187 Dresden, Germany; 4 Heinz Maier-Leibnitz Zentrum (MLZ), TU München, Lichtenbergstraße 1, D-85747 Garching, Germany; 5 Jülich Centre for Neutron Science (JCNS), Forschungszentrum Jülich GmbH, Outstation at Heinz Maier-Leibnitz Zentrum (MLZ), Lichtenbergstraße 1, D-85747 Garching, Germany; 6 Quantum Condensed Matter Division, Oak Ridge National Laboratory (ORNL), Oak Ridge, Tennessee 37831, USA; 7 Leibniz Institute for Solid State and Materials Research, IFW Dresden, Helmholtzstraße 20, D-01069 Dresden, Germany

## Abstract

Complex low-temperature-ordered states in chiral magnets are typically governed by a competition between multiple magnetic interactions. The chiral-lattice multiferroic Cu_2_OSeO_3_ became the first insulating helimagnetic material in which a long-range order of topologically stable spin vortices known as skyrmions was established. Here we employ state-of-the-art inelastic neutron scattering to comprehend the full three-dimensional spin-excitation spectrum of Cu_2_OSeO_3_ over a broad range of energies. Distinct types of high- and low-energy dispersive magnon modes separated by an extensive energy gap are observed in excellent agreement with the previously suggested microscopic theory based on a model of entangled Cu_4_ tetrahedra. The comparison of our neutron spectroscopy data with model spin-dynamical calculations based on these theoretical proposals enables an accurate quantitative verification of the fundamental magnetic interactions in Cu_2_OSeO_3_ that are essential for understanding its abundant low-temperature magnetically ordered phases.

## Abstract (web summary)

Cu_2_OSeO_3_ possesses a helical spin structure which supports a rich phase diagram of magnetic states, including a lattice of particle-like skyrmions. Here, the authors use inelastic neutron scattering to elucidate the three-dimensional magnon spectrum and underlying spin Hamiltonian of Cu_2_OSeO_3_.

## Graphical abstract

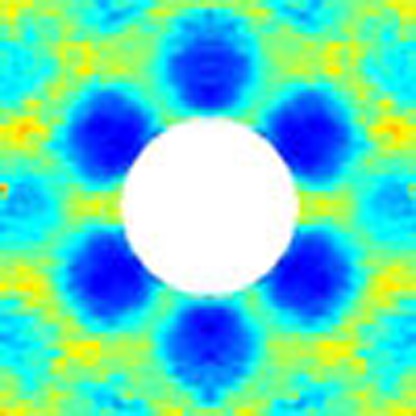

Chiral magnets form a broad class of magnetic materials in which long-range dipole interactions, magnetic frustration, or relativistic Dzyaloshinskii–Moriya (DM) interactions twist the initially ferromagnetic spin arrangement, thus leading to the formation of noncollinear incommensurate helical structures with a broken space-inversion symmetry^1^,^2^,^3^,^4^. Helical magnetic states occur in a very broad range of compounds including metals and alloys, semiconductors and multiferroics. From a fundamental point of view, the significance of such materials is due to the richness of their possible magnetic structures as compared to ordinary (commensurate and collinear) magnets. Perhaps the most prominent example is given by the topologically non-trivial long-range ordered states known as skyrmion lattices^5^,^6^,^7^.

The interest in the multiferroic ferrimagnet Cu_2_OSeO_3_ with a chiral crystal structure has been intensified in recent years after the discovery of a skyrmion-lattice phase in this system^8^, thus making it the first known insulator that exhibits a skyrmion order. It was shown soon afterwards that this skyrmion lattice can be manipulated by the application of either magnetic^9^ or electric^10^ field, and more recently also by chemical doping^11^, which indicates a delicate balance of the magnetic exchange and DM interactions with the spin anisotropy effects. Understanding the complex magnetic phase diagram of Cu_2_OSeO_3_ therefore requires detailed knowledge of the spin Hamiltonian with precise quantitative estimates of all interaction parameters, which can be obtained from measurements of the spin-excitation spectrum. Microscopic theoretical models that were recently proposed for the description of spin arrangements in Cu_2_OSeO_3_ include five magnetic exchange integrals and five anisotropic DM couplings between neighbouring 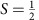 copper spins^12^,^13^,^14^,^15^,^16^, yet their experimental verification was so far limited to thermodynamic data^14^, terahertz electron spin resonance (ESR)^15^, far-infrared^17^ and Raman spectroscopy^18^ that can only probe zone-centre excitations in reciprocal space. Here we present the results of inelastic neutron scattering (INS) measurements that reveal the complete picture of magnetic excitations in Cu_2_OSeO_3_ accessible to modern neutron spectroscopy over the whole Brillouin zone and over a broad range of energies and demonstrate good quantitative agreement with spin-dynamical model calculations both in terms of magnon dispersions and dynamical structure factors.

## Results

### Crystal structure and Brillouin zone unfolding

**Figure 1:**
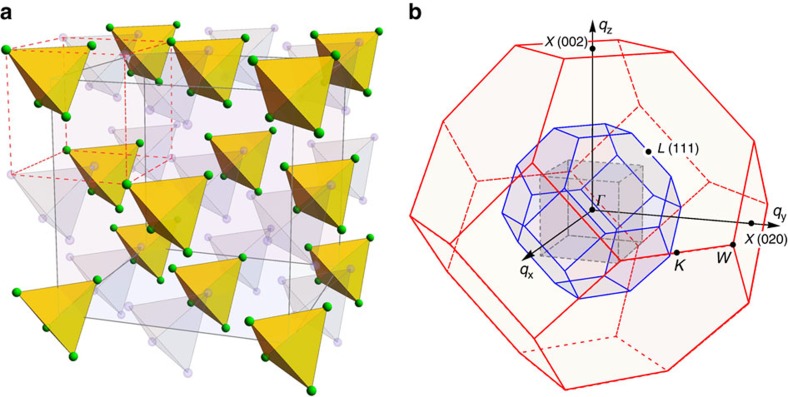
Simplified structure of the magnetic Cu sublattice in Cu_2_OSeO_3_ and Brillouin zone unfolding. (**a**) The sublattice of Cu atoms approximated by an fcc arrangement of identical tetrahedral Cu_4_ clusters, the corresponding unit cell shown with grey lines. Introduction of imaginary tetrahedra (faded colour) within the voids allows to introduce a twice smaller structural unit cell (dashed red lines) that corresponds to a half-filled fcc lattice of Cu atoms. (**b**) Brillouin zones that correspond to the original crystallographic simple-cubic unit cell with the lattice parameter *a* (inner grey cube), the fcc unit cell with lattice parameter *a* (smaller truncated octahedron, blue lines) and the fcc unit cell with lattice parameter *a*/2 (larger truncated octahedron, red lines).

The cubic copper(II)-oxoselenite Cu_2_OSeO_3_ crystallizes in a complex chiral structure with 16 formula units per unit cell (space group *P*2_1_3, lattice constant *a*=8.925 Å)^19^. This structure has been visualized, for instance, in refs 8, 14. For the discussion of magnetic properties relevant for our study, it is useful to consider only the magnetic sublattice of copper ions, which can be approximated by a face-centred cubic (fcc) lattice of identical Cu_4_ tetrahedra, as shown in Fig. 1a. This reduces the volume of the primitive unit cell fourfold with respect to the original simple-cubic structure, resulting in only four Cu atoms per primitive cell. In reciprocal space, this simplified lattice possesses a larger ‘unfolded' Brillouin zone with a volume of 4(2*π*/*a*)^3^ as shown in Fig. 1b with blue lines.

Existing theoretical models^13^,^14^,^15^ suggested that individual Cu_4_ tetrahedra represent essential magnetic building blocks of Cu_2_OSeO_3_. Because of the structural inequivalence of the copper sites within every tetrahedron, its magnetic ground state orients one of the four Cu^2+^ spins antiparallel to three others that are coupled ferromagnetically, resulting in a state with the total spin *S*=1. The interactions between neighbouring tetrahedral clusters are at least 2.5 times weaker than intra-tetrahedron couplings^13^,^14^,^15^ and lead to a ferromagnetic arrangement of their total spins below the Curie temperature, *T*_C_=57 K. In addition, weak DM interactions twist the resulting ferrimagnetic state into an incommensurate helical spin structure propagating along the 〈001〉 direction with a pitch *λ*_h_=63 nm, that is 100 times larger than the distance between nearest-neighbour tetrahedra, which corresponds to the propagation vector *k*_h_=0.010 Å^−1^ derived from small-angle neutron-scattering data^9^.

As the next step, we note that the same structure can be represented as a half-filled fcc lattice of individual Cu atoms. Indeed, if one supplements the lattice with an equal number of imaginary Cu_4_ tetrahedra by placing them within the voids of the original structure, as shown in Fig. 1a, a twice smaller fcc unit cell with lattice parameter *a*/2 can be introduced (red dashed lines). Its primitive cell contains on average one half of a copper atom and corresponds to the large ‘unfolded' Brillouin zone with a volume of 32(2*π*/*a*)^3^ that is shown with red lines in Fig. 1b. As will be seen in the following, the dynamical structure factor representing the distribution of magnon intensities in Cu_2_OSeO_3_ inherits the symmetry of this large Brillouin zone. The described unfolding procedure is therefore useful for reconstructing the irreducible reciprocal-space volume for the presentation of INS data. Throughout this paper, we will denote momentum space coordinates in reciprocal lattice units corresponding to the crystallographic simple-cubic unit cell (1 r.l.u.=2*π*/*a*), whereas high-symmetry points will be marked in accordance to the large unfolded Brillouin zone as explained in Fig. 1b. Equivalent points from higher Brillouin zones will be marked with a prime.

### Triple-axis neutron spectroscopy

**Figure 2:**
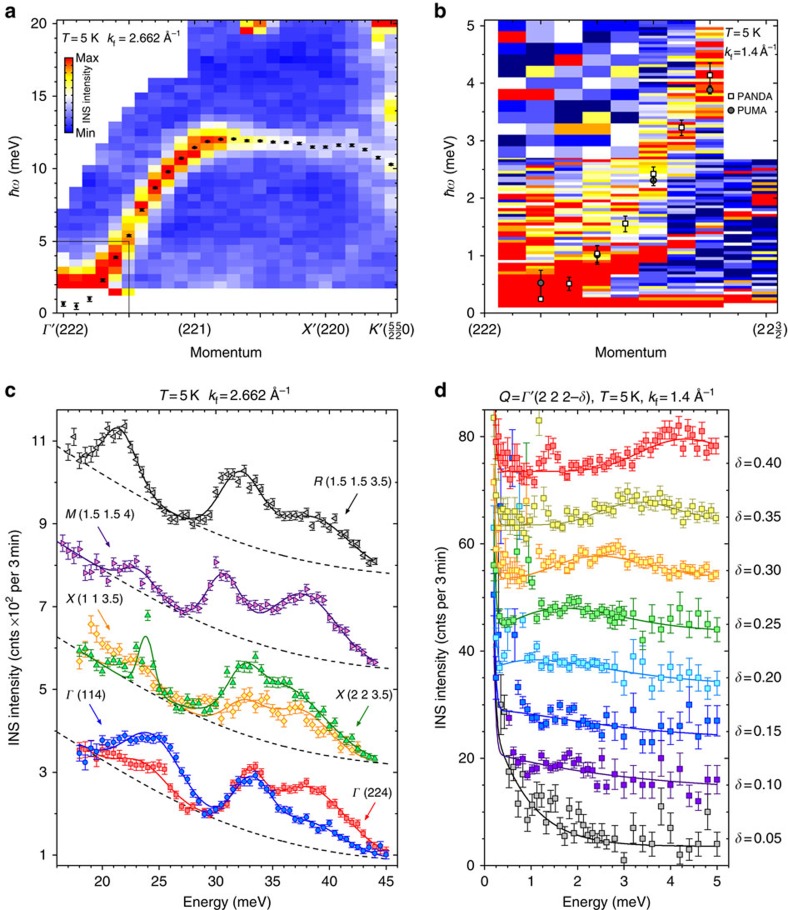
Magnon excitations in Cu_2_OSeO_3_ as measured by triple-axis neutron spectroscopy. All the presented data were measured in the low-temperature spin-spiral state (*T*=5 K). (**a**) Dispersion of the low-energy ferromagnon branch along the (00*H*) direction mapped out with the thermal-neutron spectrometer PUMA. Data points represent peak positions that resulted from fitting the energy profiles for every momentum. (**b**) The lower part of the same dispersion within the region shown by a black rectangle in **a** measured with higher resolution at the cold-neutron spectrometer PANDA. The magnon corresponds to the higher-energy broadened peak (squares), the sharp weaker peaks below it originate from spurious Bragg scattering. The peak positions from the thermal-neutron data in **a** are also shown here for comparison (grey circles). (**c**) Raw energy scans across the upper magnon bands measured at several high-symmetry points using the PUMA spectrometer. Here the spectra are grouped and marked according to the small (original crystallographic) cubic Brillouin zone. (**d**) Individual energy cuts from the cold-neutron dataset in **b** for different momenta along (2 2 2–*δ*). The fits shown with solid lines neglect the spurious peaks at the low-energy side of the spectrum. The error bars in all panels represent 1 s.d. of the count rate.

We start the presentation of our experimental results with the triple-axis spectroscopy (TAS) data shown in Fig. 2. At low energies, the spectrum is dominated by an intense Goldstone mode with a parabolic dispersion, which emanates from the *Γ*′(222) wave vector and has the highest intensity at this point, as can be best seen from the thermal-neutron data in Fig. 2a. Because this point corresponds to the centre of the large unfolded Brillouin zone, we can associate this low-energy mode with a ferromagnetic spin-wave branch anticipated from the theory for the collinear magnetically ordered state^13^. Upon moving away from the zone centre along the (001) direction, its dispersion reaches a maximum of ∼12 meV, while its intensity is reduced continuously towards the unfolded-zone boundary. Much weaker replicas of the same ferromagnon branch can be also recognized at other points with integer coordinates, like (221) or (220), as they coincide with zone centres of the original crystallographic Brillouin zone but not the unfolded one. We have fitted individual energy scans in Fig. 2a with Gaussian peak profiles to extract the measured magnon dispersion shown as black dots.

For a closer examination of the low-energy part of the ferromagnon branch around the *Γ*′(222) point, we also performed TAS measurements at a cold-neutron spectrometer providing higher energy resolution, as shown in Fig. 2b,d. Here we see a parabolically dispersing ferromagnetic spin-wave branch (squares) in good agreement with the dispersion obtained from the thermal-neutron measurements (grey circles). The data are additionally contaminated with spurious Bragg scattering that appears as a sharp straight line below the magnon peak in Fig. 2b or as a strong low-energy peak in Fig. 2d. While fitting the experimental data to extract the magnon dispersion (solid lines in Fig. 2d), this spurious peak had to be masked out. As a result, the ferromagnon mode appears to be gapless within our experimental resolution, which agrees with the earlier microwave absorption measurements, where the spin gap value of only 3 GHz (12 μeV) has been reported^20^. By fitting the experimental dispersion at low energies with a parabola, *ħω*=*Dq*^2^, where *q* is the momentum transfer along (001) measured relative to the (222) structural Bragg reflection, we evaluated the spin-wave stiffness *D*=52.6 meV Å^2^, which is comparable to that in the prototypical metallic skyrmion compound MnSi (ref. 21).

The magnetic Goldstone mode is significantly broadened in both energy and momentum and has a large intrinsic width that is considerably exceeding the instrumental resolution of ∼0.14 meV. This can be naturally explained as a result of the incommensurability of the spin-spiral structure and its deviation from the collinear ferrimagnetic order. In the helimagnetic state, the low-energy magnetic excitations are expected to split into so-called helimagnons emanating from the first-, second- and higher-order magnetic Bragg reflections^22^,^23^,^24^. The incommensurability parameter *k*_h_ is too small in our case to be resolved with a conventional triple-axis spectrometer. The typical helimagnon energy scale in Cu_2_OSeO_3_ can be estimated as 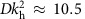 μeV, that is an order of magnitude lower than in MnSi and also lies well below the energy resolution of the instrument. As a result, multiple unresolved helimagnon branches merge into a single broadened peak as seen in Fig. 2d.

According to the theoretical calculations^13^, a higher-energy band of dispersive magnon excitations is expected between ∼25 and 40 meV. In Fig. 2c, we show several spectra measured in this energy range at a number of high-symmetry points that reveal at least three distinct peaks. Here we have grouped the spectra according to their location in the original crystallographic Brillouin zone (grey cube in Fig. 1b) to emphasize that the peak intensities are strongly affected by the dynamical structure factors, whereas their positions in energy in different Brillouin zones remain essentially the same for equivalent wave vectors. The theory anticipates that these high-energy modes represent an intricate tangle of multiple magnon branches, so that several of them may contribute to every experimentally observed peak. A direct comparison with the existing spin-dynamical calculations, therefore, appears difficult with the limited TAS data and requires a complete mapping of the momentum–energy space using time-of-flight (TOF) neutron spectroscopy that we present below.

### Time-of-flight neutron spectroscopy

**Figure 3:**
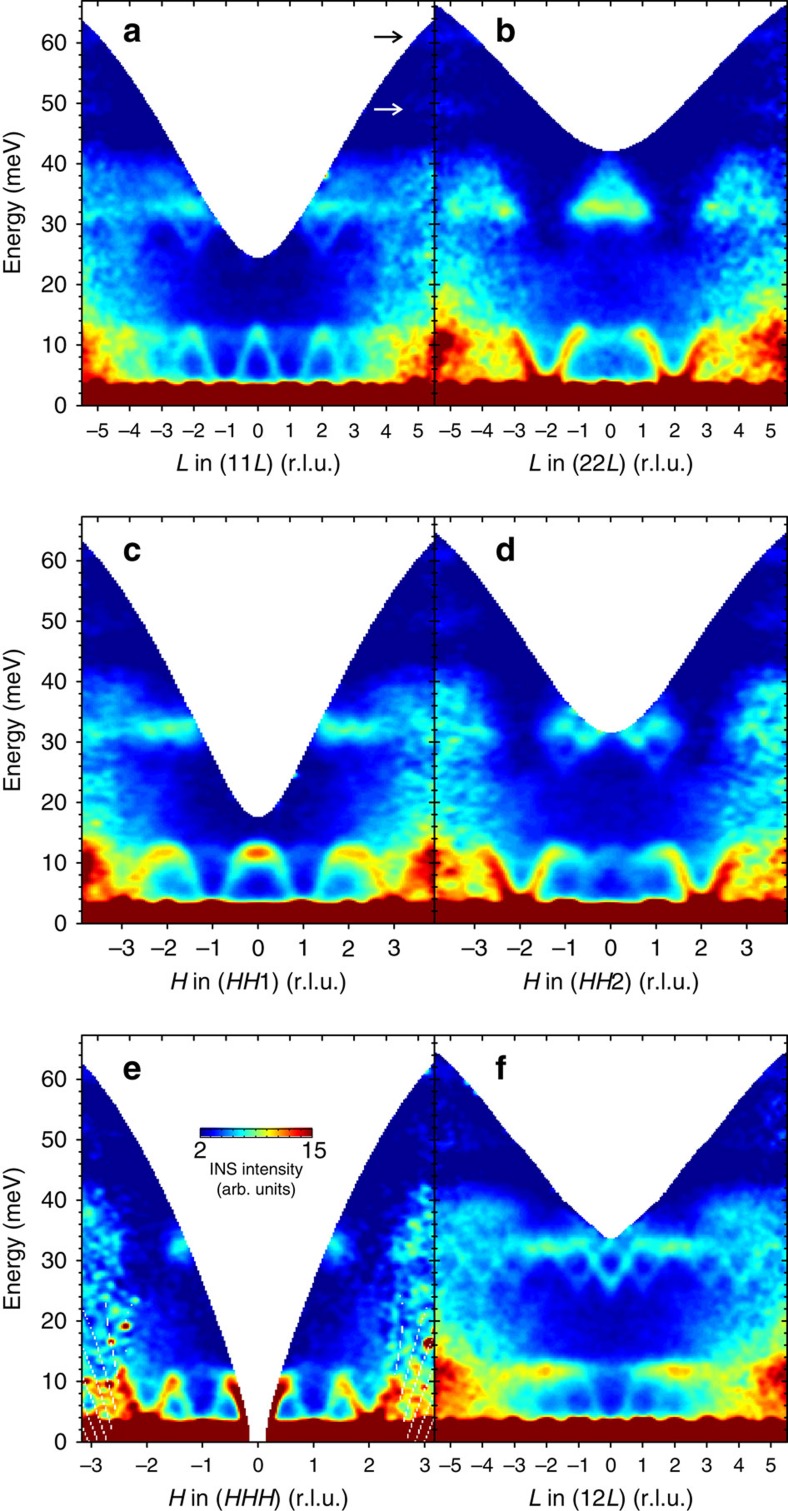
Energy–momentum cuts through the TOF data. The colour maps show INS intensity distributions along several representative high-symmetry directions: (**a**) (11*L*); (**b**) (22*L*); (**c**) (*HH*1); (**d**) (*HH*2); (**e**) (*HHH*); and (**f**) (12*L*). All the presented data were measured at the base temperature of 4.5 K. Black and white arrows mark two high-energy optical phonon modes.

The benefit of TOF neutron scattering is that it provides access to the whole four-dimensional energy–momentum space (*ħω*, **Q**) in a single measurement, which is particularly useful for mapping out complex magnetic dispersions that persist over the whole Brillouin zone. The data can be then analysed to extract any arbitrary one- or two-dimensional cut from this data set as we describe in the Methods. Figure 3 presents several typical energy–momentum cuts taken along different high-symmetry lines parallel to the (001), (110) and (111) crystallographic axes. One clearly sees both the low-energy ferromagnon band that is most intense around *Γ*′(222) and the intertwined higher-energy dispersive magnon modes between 25 and 40 meV whose intensity anticorrelates with that of the low-energy modes: it vanishes near *Γ*′(222) yet is maximized near the *X*′(220) point at the unfolded-zone boundary, where the low-energy modes are weak. The low- and high-energy modes are separated by a broad energy gap in the range ∼13–25 meV. In addition, at much higher energies one can see two relatively weak flat modes centred around 49 and 61 meV (marked with arrows in Fig. 3a). Although the theory predicts a flat magnetic mode around 54 meV (ref. 13), the fact that their intensity monotonically increases towards higher |**Q**| speaks rather in favour of optical phonons. It is likely that the 54 meV magnetic mode is far too weak compared with the phonon peaks and therefore cannot be clearly seen in our data. As the data in Fig. 3 extend over several equivalent Brillouin zones in momentum space, one can see an increase of the nonmagnetic background and a simultaneous decrease of the magnetic signal towards higher |**Q**|, resulting in a rapid reduction of the signal-to-background ratio. Therefore, in the following we restrict our analysis mainly to the irreducible part of the reciprocal space corresponding to the shortest possible wave vectors between *Γ*(000) and *Γ*′(222).

It is interesting to note how the hierarchical structure of Brillouin zones introduced in Fig. 1 is directly reflected in the magnon intensities. Along the (*HHH*) direction in Fig. 3e, the strongest spin-wave modes are seen around centres of the large unfolded zones, *Γ*(000) and *Γ*′(222), while a somewhat weaker mode appears at the *L*(111) point that coincides with the zone centre for the smaller fcc Brillouin zone (shown in blue in Fig. 1b). Other points with integer coordinates that are centres of the smallest simple-cubic zones but of none of the larger ones, such as (110) or (001), contain only much weaker replica bands that are barely seen in the data (for example, Figs 2a and 3a,c). This leads to the appearance of rather unusual features in certain cuts through the spectrum, such as the one at the (001) point in the middle of Fig. 3c, which visually resembles the bottom part of the famous hourglass-like dispersion in some transition-metal oxides^25^,^26^,^27^. However, here its intense top part stems from the nearest unfolded-zone centre *Γ*(000) that lies outside of the image plane, while the weaker downward-dispersing branches originate from the *L* points at 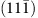 and (111), producing such a peculiar shape. The situation is even more complicated at higher energies, where the strongest well-resolved downward-pointing modes with a bottom around 26 meV are located at *W* points of the large unfolded zone, such as (102), (210) or (320), with weaker replicas shifted by the (111) or equivalent vector. Interestingly, without Brillouin zone unfolding such a particularity of the *W* points would not at all be obvious without explicit calculations of the dynamical structure factors, as they are all equivalent to the zone centre in the original crystallographic Brillouin zone notation.

**Figure 4:**
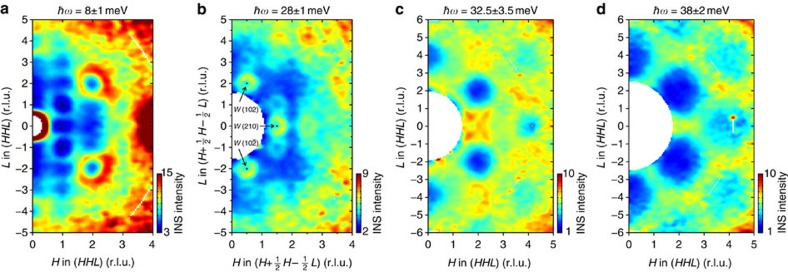
Constant-energy slices through the TOF data parallel to the (*HHL*) plane. (**a**) Along the (*HHL*) plane at *ħω*=8±1 meV cutting through the low-energy magnon band with intense rings of scattering from the ferromagnon modes around *Γ* points; (**b**) Along the offset 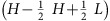 plane at *ħω*=28±1 meV through the bottom part of the upper magnon band, showing rings of intensity around *W* points; (**c**,**d**) Along the (*HHL*) plane through the upper magnon band at *ħω*=32.5±3.5 meV and 38±2 meV, respectively. In all panels, the data were integrated within ±0.15 r.l.u. from the indicated plane along the 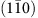 direction that is orthogonal to the plane of the figure. Corresponding energy integration ranges given above each panel are also shown in Fig. 5a with vertical bars labelled A–D.

The pattern of INS intensity in momentum space is visualized by the constant-energy slices presented in Fig. 4. All of them are oriented parallel to the (*HHL*) plane, which was horizontal in our experimental geometry. The low-energy cut integrated around 8±1 meV (Fig. 4a) passes through the origin and contains both the (222) and (111) wave vectors, where rings of scattering from the stronger and weaker magnetic Goldstone modes, respectively, can be clearly seen. The next cut at 28±1 meV in Fig. 4b is shifted by the 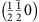 vector in such a way that it crosses the previously mentioned downward-pointing high-energy modes, which appear as small circles around *W* points. Finally, the last two panels (Fig. 4c,d) show integrated intensity in the (*HHL*) plane at energies corresponding to the middle (32.5±3.5 meV) and top (38±2 meV) of the upper magnon band, respectively. While individual magnon dispersions are intertwined at these energies and cannot be resolved separately, in both panels we observe deep minima of intensity at the *Γ*′(222), *Γ*″(400) and equivalent wave vectors. They correspond to the suppression of the dynamical structure factor at the centre of the unfolded Brillouin zone, where the low-energy ferromagnon mode, on the contrary, is most intense.

**Figure 5:**
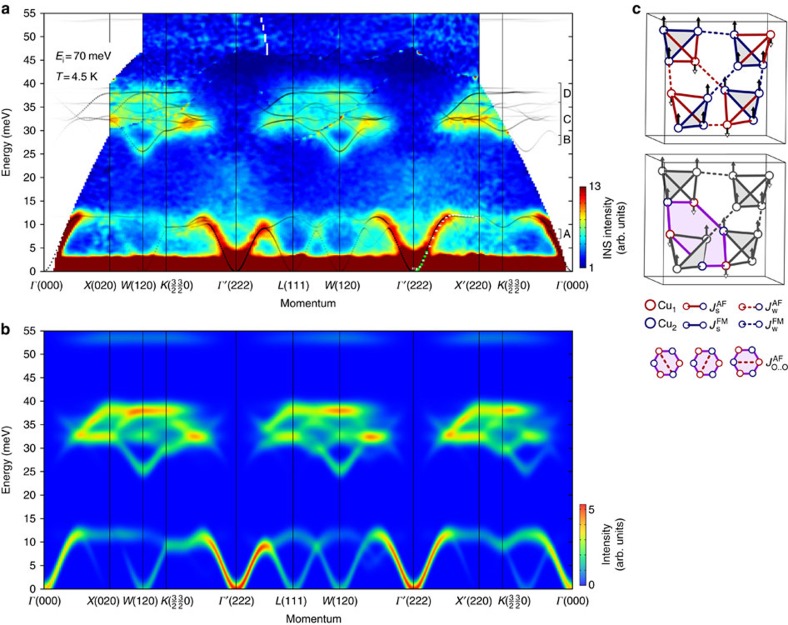
Symmetrized TOF data along high-symmetry directions. (**a**) Energy–momentum profile of the symmetrized low-temperature TOF data (*T*=4.5 K) along a polygonal path involving most of the high-symmetry directions in **Q**-space. The data within every segment have been averaged with all equivalent cuts in the same Brillouin zone. The peak positions extracted from the fits to the thermal- and cold-neutron TAS data between the *Γ*′(222) and *X*′(220) points are shown here with white and green data points. The vertical bars marked as A–D on the right-hand side of the panel show energy integration ranges of the corresponding constant-energy cuts in Fig. 4. Dotted lines show the calculated dispersions, their transparency reflecting the dynamical structure factors (calculated intensities) as described in the text. (**b**) Calculated scattering intensity presented as a colour map that was obtained by broadening the results of our spin-dynamical calculations with a Gaussian function meant to model the experimental resolution. (**c**) Exchange paths in Cu_2_OSeO_3_. Top: First-neighbour ferromagnetic (blue) and antiferromagnetic (red) couplings. Weak bonds are indicated with dashed lines. Bottom: Further-neighbour interaction 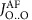 realized on the diagonals of hexagons formed by alternating Cu_1_ and Cu_2_ sites.

To get a more complete picture of magnetic excitations in Cu_2_OSeO_3_ and to compare them with the results of spin-dynamical calculations, we have symmetrized the TOF data (see Methods) and assembled energy–momentum profiles along a polygonal path involving all high-symmetry directions in **Q**-space into a single map presented in Fig. 5a. Due to the kinematic constraints, the data in the first Brillouin zone are always limited to lower energies as can be seen in Fig. 3, but have the best signal-to-noise ratio. Therefore, for the best result we also underlayed data from higher Brillouin zones to show the higher-energy part of the spectrum at equivalent wave vectors in the unfolded notation.

### Spin-dynamical calculations

Based on previous theoretical and experimental results^13^,^14^,^15^, we used a tetrahedron-factorized multi-boson method to calculate the magnon spectrum for the collinear ferrimagnetically ordered state. We consider the Heisenberg-type Hamiltonian:


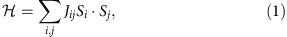


where *J*_*ij*_ denote five different kinds of exchange couplings between sites *i* and *j* that are explained in Fig. 5c. There are two strong couplings, a ferromagnetic 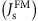 and an antiferromagnetic 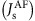 one, residing on the Cu_4_ tetrahedra that form the elementary units of our tetrahedra-factorized multiboson theory. Furthermore, there are weak ferromagnetic 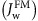 and antiferromagnetic 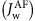 first-neighbour interactions connecting the tetrahedra. The last coupling is an antiferromagnetic longer-range interaction (*J*_O..O_) connecting Cu_1_ and Cu_2_ across the diagonals of hexagonal loops formed by alternating Cu_1_ and Cu_2_ sites. These five interactions are treated on a mean-field level, whereas the antisymmetric DM interactions are neglected for simplicity, as they would affect only the lowest frequency range of the spectrum in the vicinity of the zone centre. This same model has been discussed in detail in ref. 13, for details see also Supplementary Notes 1 and 2 with the Supplementary Table 1. Having a complete picture of magnetic excitations in the entire Brillouin zone up to high energies, we can refine the previously established interaction parameters determined from *ab-initio* calculations^14^ and ESR experiments^15^.

The values for weak interaction parameters were found to be 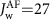 K, 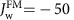 K and 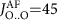 K, as defined earlier by fitting the magnetization data^14^. These parameters, in fact, provide low-lying modes in agreement with the TOF data as shown in Fig. 5a. Strong coupling constants within the tetrahedra, 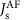 and 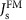, are mainly responsible for the positions of high-energy modes and govern the intra-tetrahedron excitations. We keep 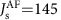 K as it was determined from fitting the ESR spectrum^15^. Modifying 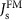 to −170 K, we can reproduce the high-energy modes in excellent agreement with experiment, as seen in Fig. 5a, where the result of the spin-dynamical calculations is shown with dotted lines (see also Supplementary Note 1 with Supplementary Figs 1 and 2).

To fully test our model, we also calculated the scattering cross section. At zero temperature this corresponds to


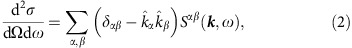


where *α*, *β*={*x*, *y*, *z*}, 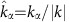, and *S*^*αβ*^(***k***, *ω*) is the dynamical spin structure factor. Here we used the original crystallographic unit cell and the corresponding cubic Brillouin zone, taking the exact positions of each copper atom within the unit cell into account. The theoretically established scattering intensity plot (artificially broadened to model the experimental resolution) is shown in Fig. 5b, demonstrating strikingly good agreement with the INS data.

## Discussion

We have presented the complete overview of spin excitations in Cu_2_OSeO_3_ throughout the entire Brillouin zone and over a broad energy range. The data reveal low-energy ferromagnetic Goldstone modes and a higher-energy band of multiple intertwined dispersive spin-wave excitations that are separated by an extensive energy gap. All the observed features can be excellently described by the previously developed theoretical model of interacting Cu_4_ tetrahedra, given that one of the strong interaction parameters, 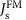 is set to a new value of −170 K. The complete list of the dominant parameters for the magnetic Hamiltonian determined here is now able to describe all the available experimental results (magnetization, ESR and INS data) simultaneously. Our model can serve as a starting point for more elaborate low-energy theories that would be able to explain the complex magnetic phase diagram of Cu_2_OSeO_3_, including the helimagnetic order and skyrmion-lattice phases, which still represents a challenge of high current interest in solid-state physics.

## Methods

### Sample preparation

The compound Cu_2_OSeO_3_ has first been synthesized by a reaction of CuO (Alfa Aesar 99.995%) and SeO_2_ (Alfa Aesar 99.999%) at 300 °C (2 days) and 600 °C (7 days) in evacuated fused silica tubes. Starting from this microcrystalline powder, single crystals of Cu_2_OSeO_3_ were then grown by chemical transport reaction using NH_4_Cl as a transport additive, which decomposes in the vapour phase into ammonia and the transport agent HCl. The reaction was performed in a temperature gradient from 575 °C (source) to 460 °C (sink), and a transport additive concentration of 1 mg cm^−3^ NH_4_Cl (Alfa Aesar 99.999%)^18^. The resulting single crystals with typical sizes of 30–60 mm^3^ were selectively characterized by magnetization, dilatometry, and X-ray diffraction measurements, showing good crystallinity and reproducible behaviour of physical properties. The crystals were then coaligned into a single mosaic sample for neutron-scattering measurements with a total mass of ∼2.5 g using a digital X-ray Laue camera.

### Triple-axis INS measurements

The measurements presented in Fig. 2 have been performed at the PUMA thermal-neutron spectrometer with a fixed final neutron wave vector *k*_f_=2.662 Å^−1^ and the PANDA cold-neutron spectrometer with *k*_f_=1.4 Å^−1^, both at the FRM-II research reactor in Garching, Germany. A pyrolytic graphite or cold beryllium filter, respectively, was installed after the sample to suppress higher order scattering contamination from the monochromator. The sample was mounted in the (*HHL*) scattering plane and cooled down with a closed-cycle refrigerator to the base temperature of ∼5 K in both experiments.

### Time-of-flight measurements

TOF data in Figs 3, 4, 5 were collected using the wide angular range chopper spectrometer ARCS^28^ at the Spallation Neutron Source, Oak Ridge National Laboratory, with the incident neutron energy set to *E*_i_=70 meV. Here the base temperature of the sample was at 4.5 K. The reciprocal space was mapped by performing a full 360° rotation of the sample about the vertical 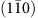 axis in steps of 1° counting ∼20 min per angle. The experimental energy resolution measured as the full width at half maximum of the elastic line was ∼2.5 meV.

### Data analysis

The TOF data were normalized to a vanadium reference measurement for detector efficiency correction, combined and transformed into energy–momentum space using the open-source MATLAB-based HORACE analysis software. Because of the high symmetry of the cubic lattice, the TOF data set could be symmetrized in order to improve statistics in the data and thereby improve the signal-to-noise ratio considerably. After the establishment of the fact, that the intensity of the INS signal follows the symmetry of the large unfolded Brillouin zone introduced in Fig. 1b, we have assumed this symmetry for the symmetrization. This means that every energy–momentum cut in Fig. 3 and every segment of Fig. 5a has been averaged with all possible equivalent cuts within the same Brillouin zone (cuts from different Brillouin zones with different |**Q**| were not averaged). For example, the *LW* or *LK* segments forming the radius and apothem of the hexagonal face of the unfolded Brillouin zone boundary, respectively, (Fig. 1b) have 48 equivalent instances along six non-parallel directions in the Brillouin zone that can be averaged. As this kind of data analysis is very time consuming and requires significant computational efforts, it still awaits to become the standard practice in neutron-scattering research.

### Software availability

The open-source MATLAB-based HORACE software package is available from http://horace.isis.rl.ac.uk.

## Additional information

**How to cite this article:** Portnichenko, P. Y. *et al*. Magnon spectrum of the helimagnetic insulator Cu_2_OSeO_3_. *Nat. Commun.* 7:10725 doi: 10.1038/ncomms10725 (2016).

## Supplementary Material

Supplementary Information Supplementary Figures 1-2, Supplementary Table 1, Supplementary Notes 1-2 and Supplementary Reference
