# Supplementary material for: Magnon spectrum of the helimagnetic insulator Cu2OSeO3
Source: Nat Commun. 2016 Feb 25;7:10725. doi: 10.1038/ncomms10725 (PMC4773425; doi:10.1038/ncomms10725)
Supplement: Supplementary Information — Supplementary Figures 1-2, Supplementary Table 1, Supplementary Notes 1-2 and Supplementary Reference [file ncomms10725-s1.pdf]

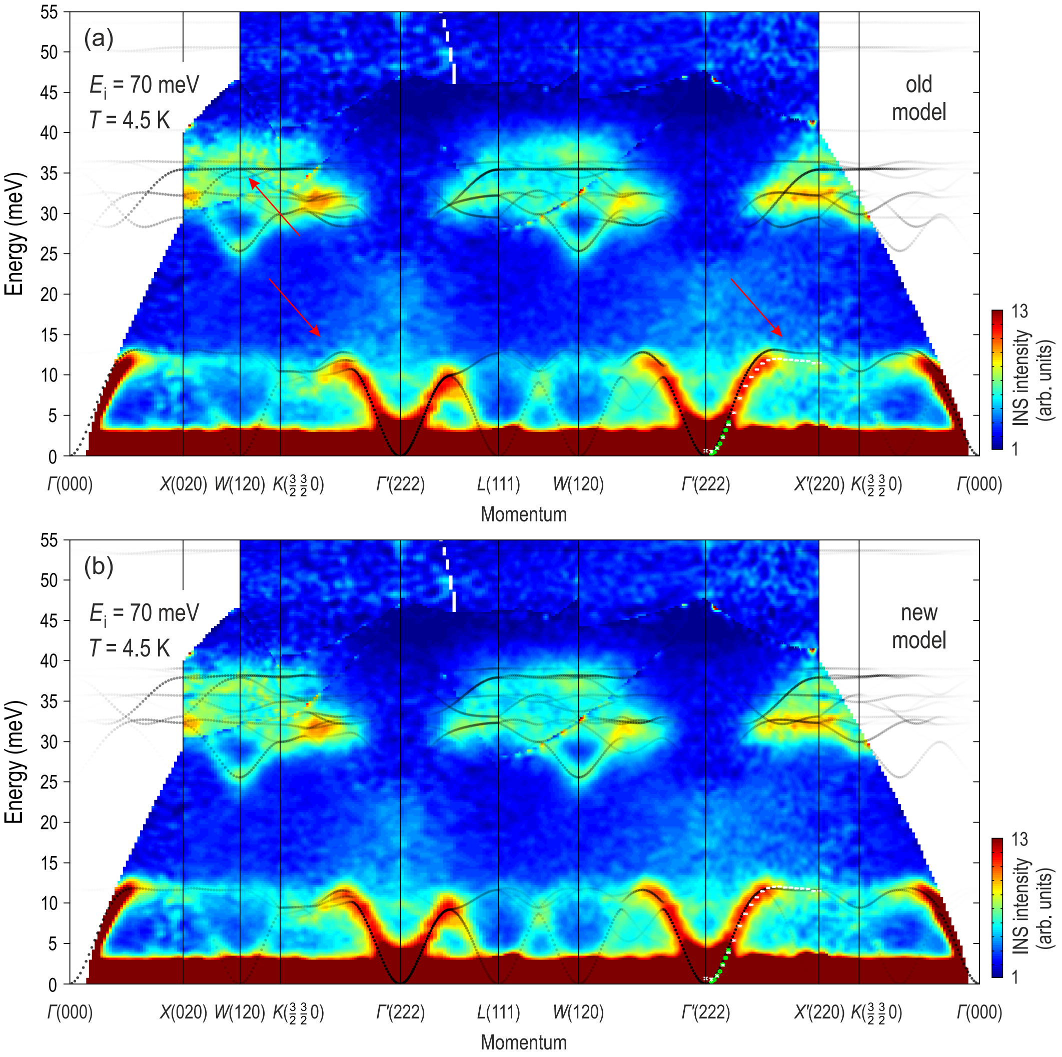


**Supplementary Figure 1 | Comparison of the old and new theoretical models with the INS data.** The same energy-momentum color map of the symmetrized low-temperature TOF data as shown in Fig. 5a of the paper, compared with the theoretically calculated dispersions (dotted lines) for two sets of exchange parameters: **a**, The original set of parameters from Ref. 1; **b**, the modified set of parameters presented in our current work, where the strong ferromagnetic exchange interaction $J_s^{FM}$ has been modified to –170 K, resulting in a better match with the experimental data.

## Supplementary Note 1

*Comparison of the theoretical models*

In the Supplementary Figure 1, we compare our time-of-flight neutron data with theoretical calculations done using the originally published exchange parameters from Ref. 1 (Supplementary Figure 1a) and the modified model with $J_s^{FM} = -170$ K (Supplementary Figure 1b) that demonstrates a significantly better agreement with the experimental data, especially in the regions marked with red arrows. In particular, in the high-energy region the old model produced a nearly dispersionless intense feature that coincided with a minimum in the observed inelastic scattering intensity, whereas in the corrected model this magnon branch is shifted towards higher energies.

In the Supplementary Figure 2, we additionally compare the experimental line profiles at the $W$ points with the corresponding simulated profiles obtained from the calculated magnon dispersions after resolution broadening (as shown in Fig. 5b in the main text). Because the covered energy range for the $W(120)$ point is only limited to lower energies, we additionally show a cut at the equivalent $W'(320)$ point which provides information about the higher-energy part of the spectrum. Both points were symmetrized with the same points in equivalent Brillouin zones obtained by applying all cyclic permutations of $(HKL)$ with possible sign changes to obtain the best signal-to-noise ratio. After that both datasets were fitted globally with a set of Gaussian peaks to separate the magnetic signal from the energy-dependent background. This magnetic signal is shown as a solid line and compared with the corresponding intensity profile from the theory.

## Supplementary Note 2

*Multiboson theory for $Cu_2OSeO_3$*

Having weak and strong interactions suggests that the elementary units are not the bare Cu ions, but the quantum mechanically (QM) entangled tetrahedra formed by four Cu sites. Therefore, we consider tetrahedra-factorized variational wave function to describe the ground sate of the system and to build upon to introduce the elementary excitations:

$$|\Psi\rangle = \prod_{t=1}^{4} |\psi\rangle_t, \tag{1}$$

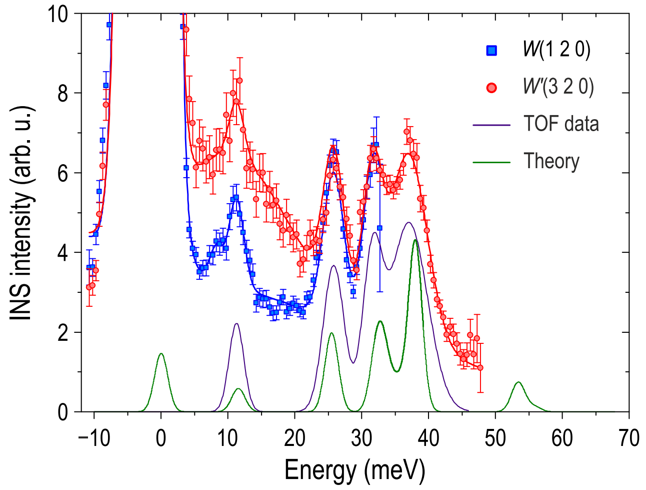


**Supplementary Figure 2 | Comparison of the measured and calculated energy profiles.** Comparison of the line profiles at the $W(120)$ and $W(320)$ points with the corresponding profiles calculated from the theoretical model after introducing a finite resolution broadening of the magnon peaks. Along with the experimental data (blue and red symbols), we additionally show the result of their fitting with a set of Gaussian peaks (blue solid line) in order to separate them from the energy-dependent background and the model curve obtained from the theory (green line).

where $|\psi\rangle_t$ is a QM state in the $2^4 = 16$ dimensional local Hilbert space of the strong tetrahedron $t$. This 16-dimensional Hilbert space can be reduced according to the $C_{3v}$ local symmetry of the distorted tetrahedron (built of one $Cu_1$ and three $Cu_2$ sites) as well as the SU(2) rotational symmetry. Such classification proves to be useful in labelling our states and establishing their mixing without elaborate calculations. Supplementary Table 1 lists the symmetry classified eigenbasis of a strong tetrahedron.

The ground state is an $A_1$ triplet separated with a large energy gap of ∼280 K from the rest of the tetrahedron spectrum. A finite weak interaction between tetrahedra mixes the multiplets, i.e. states with different total spin $S$. The local symmetry, however, remains $C_{3v}$, and the $z$ component of the spin remains a good quantum number. Thus the $A_1$ triplet can only mix with the $A_1$ quintet. Furthermore, if we select the $S^z = 1$ state as the ground state, purely for sake of simplicity, the state $|1,1,A_1\rangle$ couples solely to $|2,1,A_1\rangle$ in the tetrahedra-factorized picture. The ground state in the tetrahedra mean-field case reads

$$|\psi\rangle_t = \cos\frac{\alpha}{2}|1,1,A_1\rangle_t + \sin\frac{\alpha}{2}|2,1,A_1\rangle. \tag{2}$$

The variational parameter $\alpha$ controls the length of local spins in the following manner:

$$\langle S_1^z\rangle = -\frac{1}{4}\left(\cos\alpha + \sqrt{3}\sin\alpha\right) \text{ and } \langle S_2^z\rangle = \frac{1}{3}\left(1 - \langle S_1^z\rangle\right). \tag{3}$$

Note that for any $\alpha$ the total magnetization per tetrahedron remains $\langle S_1^z\rangle + 3\langle S_2^z\rangle = 1$, i.e. we remain in the one-half plateau phase regardless the value of the variational parameter $\alpha$. In the limit $\alpha = 0$ we recover the decoupled case with the local moments $\langle S_1^z\rangle = 1/4$ and $\langle S_2^z\rangle = 5/12$. In the coupled case for coupling values $J_s^{AF} = 145$ K, $J_s^{FM} = -170$ K, $J_w^{AF} = 27$ K, $J_w^{FM} = -50$ K, and $J_{O..O}^{AF} = 45$ K, the variational parameter takes the value $\alpha = 0.38191$ and the local moments become $\langle S_1^z\rangle = -0.39337$ and $\langle S_2^z\rangle = 0.464457$.

To include quantum fluctuations and describe the dynamical properties, we use multiboson theory — a generalizisation of the standard spin-wave approach. As a first step, we introduce the boson operators $a^\dagger_{n,t}$ that create the $n^{\text{th}}$ state ($n = 1..16$) of a tetrahedron $t$ ($t = 1..4$). As each tetrahedron can only be in one of its 16 states, the bosons must satisfy the constraint

$$\sum_{n=1}^{16} a^\dagger_{n,t} a_{n,t} = 1. \tag{4}$$

The bosonic representation of the spin operator $S_j^\alpha$, where $j$ denotes a given copper site, is too excessive to show here. Nonetheless, some operators acquire a simpler form, such as the intra-tetrahedron Hamiltonian and the total magnetization $S^z = \sum_{i=1-4} S_i^z$, which are diagonal in this basis:

$$\mathcal{H}_t = E_{1,A_1}\sum_{m=\bar{1},0,1} a^\dagger_{1,m,A_1}a_{1,m,A_1} + E_{0,E}\sum_{E=E_1,E_2} a^\dagger_{0,0,E}a_{0,0,E} + E_{2,A_1}\sum_{m=\bar{2},\dots,2} a^\dagger_{1,m,A_1}a_{1,m,A_1} + E_{1,E}\sum_{\substack{m=\bar{1},0,1\\E=E_1,E_2}} a^\dagger_{1,m,E}a_{1,m,E}, \tag{5}$$

$$S_t^z = \sum m\, a^\dagger_{S,m,R}a_{S,m,R}. \tag{6}$$

The spin raising operator of a tetrahedron $t$ can be written as

$$S_t^+ = \sum \sqrt{S(S+1) - m(m+1)}\,a^\dagger_{S,m+1,R}a_{S,m,R}, \tag{7}$$

where the sum is over the 16-dimensional tetrahedron basis, and the spin lowering operator is its hermitian conjugate.

After rewriting our spin operators in terms of boson operators $a^\dagger_{n,t}$, we transform out the boson that creates the ground state to get a Hamiltonian containing only the excitations. Then we solve this spin-wave Hamiltonian using the Bogoliubov transformation to obtain the spectrum. Our ground state is expressed in Supplementary Equations (1) and (2). Let $a^\dagger_{1,t}$ denote the boson that creates $|\psi\rangle_t$ at a tetrahedron $t$. The orthogonal bosons will represent the 15 local excitations on each tetrahedron. Thus alltogether we have $4 \times 15 = 60$ excitations. To eliminate $a^\dagger_{1,t}$ we use the above constraint and perform the following substitution

$$a^\dagger_{1,t} \to 1 - \frac{1}{2}\sum_{n>1} a^\dagger_{n,t}a_{n,t} \text{ and } a_{1,t} \to 1 - \frac{1}{2}\sum_{n>1} a^\dagger_{n,t}a_{n,t}. \tag{8}$$

In the linearized multiboson theory we only keep second order boson terms in the resulting Hamiltonian. Therefore, the multiboson Hamiltonian, now containing only the excitation

**Supplementary Table 1 |** The basis of a (strong) tetrahedron classified according to the spin rotational SU(2) and geometrical $C_{3v}$ symmetries.

| irrep | state | notation | $a_n^\dagger$ boson | energy |
|---|---|---|---|---|
| $A_1 \otimes \mathscr{D}^{(1)}$ | $\frac{1}{2\sqrt{3}}\left(\vert\Downarrow\downarrow\downarrow\uparrow\rangle + \vert\Downarrow\downarrow\uparrow\downarrow\rangle + \vert\Downarrow\uparrow\downarrow\downarrow\rangle - 3\vert\Uparrow\downarrow\downarrow\downarrow\rangle\right)$ | $\vert 1,\bar{1}\rangle_{A_1}$ | $a^\dagger_{1,\bar{1},A_1}$ | $E_{1,A_1} = -\frac{5}{4}J_s^{AF} + \frac{3}{4}J_s^{FM}$ |
| | $\frac{1}{\sqrt{6}}\left(\vert\Downarrow\downarrow\uparrow\uparrow\rangle + \vert\Downarrow\uparrow\downarrow\uparrow\rangle + \vert\Downarrow\uparrow\uparrow\downarrow\rangle - \vert\Uparrow\downarrow\downarrow\uparrow\rangle - \vert\Uparrow\downarrow\uparrow\downarrow\rangle - \vert\Uparrow\uparrow\downarrow\downarrow\rangle\right)$ | $\vert 1,0\rangle_{A_1}$ | $a^\dagger_{1,0,A_1}$ | |
| | $\frac{1}{2\sqrt{3}}\left(3\vert\Downarrow\uparrow\uparrow\uparrow\rangle - \vert\Uparrow\downarrow\uparrow\uparrow\rangle - \vert\Uparrow\uparrow\downarrow\uparrow\rangle - \vert\Uparrow\uparrow\uparrow\downarrow\rangle\right)$ | $\vert 1,1\rangle_{A_1}$ | $a^\dagger_{1,1,A_1}$ | |
| $E \otimes \mathscr{D}^{(0)}$ | $\frac{1}{2\sqrt{3}}\left(2\vert\Downarrow\downarrow\uparrow\uparrow\rangle - \vert\Downarrow\uparrow\downarrow\uparrow\rangle - \vert\Downarrow\uparrow\uparrow\downarrow\rangle - \vert\Uparrow\downarrow\downarrow\uparrow\rangle - \vert\Uparrow\downarrow\uparrow\downarrow\rangle + 2\vert\Uparrow\uparrow\downarrow\downarrow\rangle\right)$ | $\vert 0,0\rangle_E$ | $a^\dagger_{0,0,E(1)}$ | $E_{0,E} = -\frac{3}{4}J_s^{AF} - \frac{3}{4}J_s^{FM}$ |
| | $\frac{1}{2}\left(\vert\Downarrow\uparrow\downarrow\uparrow\rangle - \vert\Downarrow\uparrow\uparrow\downarrow\rangle - \vert\Uparrow\downarrow\downarrow\uparrow\rangle + \vert\Uparrow\downarrow\uparrow\downarrow\rangle\right)$ | | $a^\dagger_{0,0,E(2)}$ | |
| $A_1 \otimes \mathscr{D}^{(2)}$ | $\vert\Downarrow\downarrow\downarrow\downarrow\rangle$ | $\vert 2,\bar{2}\rangle_{A_1}$ | $a^\dagger_{2,\bar{2},A_1}$ | $E_{2,A_1} = \frac{3}{4}J_s^{AF} + \frac{3}{4}J_s^{FM}$ |
| | $\frac{1}{2}\left(\vert\Downarrow\downarrow\downarrow\uparrow\rangle + \vert\Downarrow\downarrow\uparrow\downarrow\rangle + \vert\Downarrow\uparrow\downarrow\downarrow\rangle + \vert\Uparrow\downarrow\downarrow\downarrow\rangle\right)$ | $\vert 2,\bar{1}\rangle_{A_1}$ | $a^\dagger_{2,\bar{1},A_1}$ | |
| | $\frac{1}{\sqrt{6}}\left(\vert\Downarrow\downarrow\uparrow\uparrow\rangle + \vert\Downarrow\uparrow\downarrow\uparrow\rangle + \vert\Downarrow\uparrow\uparrow\downarrow\rangle + \vert\Uparrow\downarrow\downarrow\uparrow\rangle + \vert\Uparrow\downarrow\uparrow\downarrow\rangle + \vert\Uparrow\uparrow\downarrow\downarrow\rangle\right)$ | $\vert 2,0\rangle_{A_1}$ | $a^\dagger_{2,0,A_1}$ | |
| | $\frac{1}{2}\left(\vert\Downarrow\uparrow\uparrow\uparrow\rangle + \vert\Uparrow\downarrow\uparrow\uparrow\rangle + \vert\Uparrow\uparrow\downarrow\uparrow\rangle + \vert\Uparrow\uparrow\uparrow\downarrow\rangle\right)$ | $\vert 2,1\rangle_{A_1}$ | $a^\dagger_{2,1,A_1}$ | |
| | $\vert\Uparrow\uparrow\uparrow\uparrow\rangle$ | $\vert 2,2\rangle_{A_1}$ | $a^\dagger_{2,2,A_1}$ | |
| $E \otimes \mathscr{D}^{(1)}$ | $\frac{1}{\sqrt{6}}\left(\vert\Downarrow\downarrow\downarrow\uparrow\rangle + \vert\Downarrow\downarrow\uparrow\downarrow\rangle - 2\vert\Downarrow\uparrow\downarrow\downarrow\rangle\right)$ | $\vert 1,\bar{1}\rangle_E$ | $a^\dagger_{1,\bar{1},E(1)}$ | $E_{1,E} = \frac{1}{4}J_s^{AF} - \frac{3}{4}J_s^{FM}$ |
| | $\frac{1}{\sqrt{2}}\left(\vert\Downarrow\downarrow\downarrow\uparrow\rangle - \vert\Downarrow\downarrow\uparrow\downarrow\rangle\right)$ | | $a^\dagger_{1,\bar{1},E(2)}$ | |
| | $\frac{1}{2\sqrt{3}}\left(-2\vert\Downarrow\downarrow\uparrow\uparrow\rangle + \vert\Downarrow\uparrow\downarrow\uparrow\rangle + \vert\Downarrow\uparrow\uparrow\downarrow\rangle - \vert\Uparrow\downarrow\downarrow\uparrow\rangle - \vert\Uparrow\downarrow\uparrow\downarrow\rangle + 2\vert\Uparrow\uparrow\downarrow\downarrow\rangle\right)$ | $\vert 1,0\rangle_E$ | $a^\dagger_{1,0,E(1)}$ | |
| | $\frac{1}{2}\left(\vert\Downarrow\uparrow\downarrow\uparrow\rangle - \vert\Downarrow\uparrow\uparrow\downarrow\rangle + \vert\Uparrow\downarrow\downarrow\uparrow\rangle - \vert\Uparrow\downarrow\uparrow\downarrow\rangle\right)$ | | $a^\dagger_{1,0,E(2)}$ | |
| | $\frac{1}{\sqrt{6}}\left(-2\vert\Uparrow\downarrow\uparrow\uparrow\rangle + \vert\Uparrow\uparrow\downarrow\uparrow\rangle + \vert\Uparrow\uparrow\uparrow\downarrow\rangle\right)$ | $\vert 1,1\rangle_E$ | $a^\dagger_{1,1,E(1)}$ | |
| | $\frac{1}{\sqrt{2}}\left(\vert\Uparrow\uparrow\downarrow\uparrow\rangle - \vert\Uparrow\uparrow\uparrow\downarrow\rangle\right)$ | | $a^\dagger_{1,1,E(2)}$ | |

boson operators, has zero-order boson terms, i.e. constant terms $\mathscr{H}^{(0)}$, linear terms $\mathscr{H}^{(1)}$, containing single-boson operators, and two-boson operator terms $\mathscr{H}^{(2)}$. $\mathscr{H}^{(0)}$ corresponds to the tetrahedral mean-field ground state energy, $\mathscr{H}^{(1)}$ is identically zero when the variational wave function minimizes the energy, and the quadratic $\mathscr{H}^{(2)}$ terms describe the boson hopping processes, providing the spectrum.

In particular, $\mathscr{H}^{(2)}$ takes the following form:

$$\mathscr{H}^{(2)} = \frac{1}{2}\begin{pmatrix} \mathbf{A}_\mathbf{k}^\dagger \\ \mathbf{A}_{-\mathbf{k}} \end{pmatrix}^T \begin{pmatrix} M & N \\ N^\dagger & M \end{pmatrix} \begin{pmatrix} \mathbf{A}_\mathbf{k} \\ \mathbf{A}_{-\mathbf{k}}^\dagger \end{pmatrix} \tag{9}$$

where $M = M^\dagger$ and $N = N^T$ are $60 \times 60$ matrices and the vector $\mathbf{A}_\mathbf{k}^\dagger$ contains the 60 excitations of the unit cell (15 per tetrahedron), namely 60 bosons $a_{n,t}^\dagger$ with $n = 2, \ldots, 16$ and $t = 1, \ldots, 4$:

$$\mathbf{A}_\mathbf{k}^\dagger = \left(a^\dagger_{\mathbf{k},2,1}, \ldots, a^\dagger_{\mathbf{k},16,4}\right), \tag{10}$$

The equation of motion reads

$$i\dot{a}_{n,t}(\mathbf{k}) = [a_{n,t}(\mathbf{k}), \mathscr{H}^{(2)}] = \omega_{n,t}(\mathbf{k})a_{n,t}(\mathbf{k}). \tag{11}$$

In order to find the linear combinations of bosons that diagonalize $\mathscr{H}^{(2)}$ we need to solve the eigenvalue problem

$$\begin{pmatrix} M & N \\ -N^\dagger & -M \end{pmatrix}^T \begin{pmatrix} \mathbf{u}_\mu \\ \mathbf{v}_\mu \end{pmatrix} = \omega_\mu \begin{pmatrix} \mathbf{u}_\mu \\ \mathbf{v}_\mu \end{pmatrix} \tag{12}$$

The physical eigenvectors (with positive eigenvalues) are associated with the states that diagonalize the Hamiltonian $\mathscr{H}^{(2)}$:

$$\alpha^\dagger_{\mu,\mathbf{k}} = \mathbf{u}_\mu \cdot \mathbf{A}_\mathbf{k}^\dagger + \mathbf{v}_\mu \cdot \mathbf{A}_{-\mathbf{k}} \tag{13}$$

Then $\left[\mathscr{H}^{(2)}, \alpha_\mu^\dagger\right] = \omega_\mu \alpha_\mu^\dagger$, and the bosons $\alpha_\mu^\dagger$ satisfy the commutation relation $\left[\alpha_\mu, \alpha_\nu^\dagger\right] = \delta_{\mu\nu}$. The Hamiltonian $\mathscr{H}^{(2)}$ becomes diagonal:

$$\mathscr{H}^{(2)} = \sum_{\mu=1}^{60} \omega_\mu \left(\alpha_\mu^\dagger \alpha_\mu + \frac{1}{2}\right). \tag{14}$$

## Supplementary References


[1] Romhányi, J., van den Brink, J. & Rousochatzakis, I. *Entangled tetrahedron ground state and excitations of the magnetoelectric skyrmion material $Cu_2OSeO_3$*. Phys. Rev. B **90**, 140404(R) (2014).
